# Effectiveness of a Digital Cognitive Behavior Therapy–Guided Self-Help Intervention for Eating Disorders in College Women: A Cluster Randomized Clinical Trial

**DOI:** 10.1001/jamanetworkopen.2020.15633

**Published:** 2020-08-31

**Authors:** Ellen E. Fitzsimmons-Craft, C. Barr Taylor, Andrea K. Graham, Shiri Sadeh-Sharvit, Katherine N. Balantekin, Dawn M. Eichen, Grace E. Monterubio, Neha J. Goel, Rachael E. Flatt, Anna M. Karam, Marie-Laure Firebaugh, Corinna Jacobi, Booil Jo, Mickey T. Trockel, Denise E. Wilfley

**Affiliations:** 1 Department of Psychiatry, Washington University School of Medicine, St Louis, Missouri; 2 Department of Psychiatry and Behavioral Sciences, Stanford University School of Medicine, Stanford, California; 3 Center for m^2^Health, Palo Alto University, Palo Alto, California; 4 Department of Medical Social Sciences, Northwestern University, Chicago, Illinois; 5 Interdisciplinary Center, Baruch Ivcher School of Psychology, Herzliya, Israel; 6 Department of Exercise and Nutrition Sciences, University at Buffalo, Buffalo, New York; 7 Department of Pediatrics, University of California, San Diego, San Diego; 8 Department of Psychology, Virginia Commonwealth University, Richmond; 9 Institute for Inclusion, Inquiry, and Innovation (iCubed), Virginia Commonwealth University, Richmond; 10 Department of Psychology and Neurosciences, University of North Carolina at Chapel Hill, Chapel Hill; 11 Institute of Clinical Psychology and Psychotherapy, Technische Universität, Dresden, Germany

## Abstract

**Importance:**

Eating disorders (EDs) are common, serious psychiatric disorders on college campuses, yet most affected individuals do not receive treatment. Digital interventions have the potential to bridge this gap.

**Objective:**

To determine whether a coached, digital, cognitive behavior therapy (CBT) intervention improves outcomes for college women with EDs compared with referral to usual care.

**Design, Setting, and Participants:**

This cluster randomized trial was conducted from 2014 to 2018 at 27 US universities. Women with binge-purge EDs (with both threshold and subthreshold presentations) were recruited from enrolled universities. The 690 participants were followed up for up to 2 years after the intervention. Data analysis was performed from February to September 2019.

**Interventions:**

Universities were randomized to the intervention, Student Bodies–Eating Disorders, a digital CBT-guided self-help program, or to referral to usual care.

**Main Outcomes and Measures:**

The main outcome was change in overall ED psychopathology. Secondary outcomes were abstinence from binge eating and compensatory behaviors, as well as ED behavior frequencies, depression, anxiety, clinical impairment, academic impairment, and realized treatment access.

**Results:**

A total of 690 women with EDs (mean [SD] age, 22.12 [4.85] years; 414 [60.0%] White; 120 [17.4%] Hispanic; 512 [74.2%] undergraduates) were included in the analyses. For ED psychopathology, there was a significantly greater reduction in the intervention group compared with the control group at the postintervention assessment (β [SE], −0.44 [0.10]; *d* = −0.40; *t*_1387_ = −4.23; *P* < .001), as well as over the follow-up period (β [SE], −0.39 [0.12]; *d* = −0.35; *t*_1387_ = −3.30; *P* < .001). There was not a significant difference in abstinence from any ED behaviors at the postintervention assessment (odds ratio, 1.48; 95% CI, 0.48-4.62; *P* = .50) or at follow-up (odds ratio, 1.51; 95% CI, 0.63-3.58; *P* = .36). Compared with the control group, the intervention group had significantly greater reductions in binge eating (rate ratio, 0.82; 95% CI, 0.70-0.96; *P* = .02), compensatory behaviors (rate ratio, 0.68; 95% CI, 0.54-0.86; *P* < .001), depression (β [SE], −1.34 [0.53]; *d* = −0.22; *t*_1387_ = −2.52; *P* = .01), and clinical impairment (β [SE], −2.33 [0.94]; *d* = −0.21; *t*_1387_ = −2.49; *P* = .01) at the postintervention assessment, with these gains sustained through follow-up for all outcomes except binge eating. Groups did not differ in terms of academic impairment. The majority of intervention participants (318 of 385 participants [83%]) began the intervention, whereas only 28% of control participants (76 of 271 participants with follow-up data available) sought treatment for their ED (odds ratio, 12.36; 95% CI, 8.73-17.51; *P* < .001).

**Conclusions and Relevance:**

In this cluster randomized clinical trial comparing a coached, digital CBT intervention with referral to usual care, the intervention was effective in reducing ED psychopathology, compensatory behaviors, depression, and clinical impairment through long-term follow-up, as well as realizing treatment access. No difference was found between the intervention and control groups for abstinence for all ED behaviors or academic impairment. Given its scalability, a coached, digital, CBT intervention for college women with EDs has the potential to address the wide treatment gap for these disorders.

**Trial Registration:**

ClinicalTrials.gov Identifier: NCT02076464

## Key Points

**Question:**

Does a coached, digital, cognitive behavior therapy (CBT) intervention result in improved outcomes among college women with eating disorders (EDs) compared with referral to usual care?

**Findings:**

In this cluster randomized clinical trial that included 690 women with binge-purge EDs from 27 US universities, the digital CBT intervention was superior to referral to usual care in decreasing ED psychopathology, compensatory behaviors, depression, and clinical impairment through long-term follow-up, as well as in realized treatment access. There was no difference in abstinence from all ED behaviors or academic impairment between groups.

**Meaning:**

These results support the efficacy of a coached, digital, CBT intervention for college women with EDs, which has the potential to bridge the treatment gap for this problem.

## Teaser

This cluster randomized clinical trial examines whether a coached, digital, cognitive behavior therapy intervention improves outcomes for college women with eating disorders compared with referral to usual care.

## Introduction

Eating disorders (EDs) are severe psychiatric disorders associated with high morbidity and mortality, marked impairment, and poor quality of life.^1,2^ College campuses are faced with an elevated prevalence of EDs, with 13.5% of US college women and 3.6% of US college men affected.^3^ Notably, 95% of first-time cases occur by age 25 years,^4^ highlighting the importance of intervention with this group. However, fewer than 20% of students with EDs report receiving treatment.^3,5^

Inadequacies in care delivery are associated with prolonged illness, poorer prognosis, and greater relapse, highlighting the need for improved modalities for screening and intervention, particularly in the at-risk group of college students.^6,7^ Current treatment delivery efforts for EDs on college campuses are hindered by factors such as limited counseling center capacity and access to evidence-based treatments.^6,7,8,9^ College students report additional barriers, including lack of time and stigma.^10^ Digital technologies, highlighted as the future of psychiatry,^11^ have the potential to improve mental health care on college campuses by overcoming barriers.^12^ Furthermore, online screens have been developed that can identify individuals with a possible ED, who can then be offered services.^13,14^ However, to date, there have been no large-scale studies in college populations that have evaluated the effects of a digital intervention for treating EDs, linked with an online screen.

The aim of the current study was to test the hypothesis that a digital cognitive behavioral therapy (CBT)–guided self-help program, Student Bodies–Eating Disorders (SB-ED), would significantly reduce ED psychopathology in college women screening positive for an ED (excluding anorexia nervosa), compared with referral to usual care. We focused on women because of the higher prevalence of these problems in women vs men.^3^ Secondary aims were to test the hypotheses that SB-ED, vs referral to usual care, would increase abstinence from all ED behaviors, reduce ED behaviors (ie, binge eating and compensatory behaviors), depression, anxiety, ED-associated clinical impairment, and academic impairment, and increase realized treatment access.

## Methods

### Participants and Procedure

We recruited US universities for participation in this cluster randomized clinical trial. Participants were female students at participating universities, aged 18 years or older, who completed an online EDs screen and screened positive for a *Diagnostic and Statistical Manual of Mental Disorders* (Fifth Edition)^2^ ED (except anorexia nervosa, which requires more intensive medical monitoring) using the Stanford-Washington University ED Screen^14^ by endorsing 6 or more episodes of binge eating, vomiting, and/or laxative or diuretic use in the past 3 months. The Stanford-Washington University ED Screen has high sensitivity and specificity for ED cases vs face-to-face interview.^14^ Participants also indicated their race (selecting all that applied) and ethnicity on the screen using prespecified options (although an *other* race option could be selected and defined).

Students were recruited using campus-specific recruitment strategies, developed collaboratively with campus stakeholders, including use of email, flyers, presentations, social media, and counseling or health center staff offering the study to individuals in need. For more information, see Fitzsimmons-Craft et al.^13^

All participating universities either required their own institutional review board approval or deferred to the institutional review board of record, which approved all study procedures. Informed consent was obtained online before screening. Upon screen completion, eligible participants who agreed to participate in the trial were asked to complete online assessments at baseline, at a postintervention assessment (occurring 8 months after baseline), and at 1-year and 2-year follow-up assessments. Participants were remunerated with a $10 gift card for completion of the baseline, postintervention, and 1-year assessments and with a $20 gift card for completion of the 2-year assessment. Data were managed by the study’s Data Coordinating Center.

This report follows the Consolidated Standards of Reporting Trials (CONSORT) reporting guideline. See Supplement 1 for the full protocol.

### Study Conditions

Participants were randomized at the university level to either intervention or control. Participants were provided information on how to access their assigned condition immediately upon screen completion and were reminded of this information 1 week later via email.

#### Intervention

SB-ED is a digital, guided self-help, CBT intervention. SB-ED is part of the Healthy Body Image Program, an online platform for screening and tailored intervention for college students at risk for or with EDs.^15,16,17^ The intervention covers the core components of CBT for EDs,^18^ including reducing ED behaviors (eg, via self-monitoring and regular eating), improving body image, regulating emotions, addressing shape checking and avoidance, challenging negative thoughts, and preventing relapse. The program includes psychoeducational content, as well as meal planning and tracking tools, self-monitoring logs, and other interactive tools (eg, texting platform facilitating coach-user communication). Users were provided access for 8 months, and each user was assigned a personal coach (see eTable 1 in Supplement 2 for an outline of final version of the intervention).

The program was hosted and maintained by a private company (Lantern). In year 1 of the trial, the program was offered in a traditional web-based format, using longer, weekly sessions. Because of the lower engagement in year 1 than expected and user-experience designers’ impressions of changes that could improve engagement, in years 2 and 3 of the trial, the program was redesigned to comprise 40 shorter, core sessions requiring approximately 10 minutes each. This version covered the same content in the year 1 version of the program and was offered via iPhone (Apple) mobile telephone application (app) in addition to web access. These changes were designed to facilitate user experience improvements, but the core intervention principles remained the same.^19,20^

Coaches were psychology doctoral students, social work masters students, study staff, or postdoctoral fellows, working at 1 of the universities overseeing the trial and were under the supervision of a clinical psychologist. Coaches used a clinical management dashboard to efficiently monitor multiple users. The dashboard provided information on users’ goals and intervention use, as well as the ability to message users. Coaches underwent extensive training, including in CBT for EDs,^18^ motivational interviewing, key tenets of effective digital coaching, and technical training. Coaches were responsible for providing timely messages to users, supporting them in making changes, and for providing ongoing feedback on progress and symptom changes. In addition, in years 2 and 3, in an effort to further increase usability, coaches offered up to 2 optional 20-minute telephone calls at the beginning and end of users’ time in the program. The first call was intended to build rapport, establish goals, and address barriers to use. The second call was intended to review progress and relapse prevention strategies. Clinical supervisors audited coaches’ messages regularly and each week reviewed the correspondence between 2 coaches and all of their active users, providing feedback as needed. All coaches participated in weekly group supervision.

#### Control

Upon completion of the baseline assessment, participants assigned to control received written feedback encouraging them to seek evaluation and/or treatment at their university’s counseling center. Participants were provided with specific information on how to make an appointment at their respective center.

### Randomization

The target university enrollment was 28, assuming that up to 2 universities would drop, leaving 26 participating. A blinded analyst performed randomization by randomly distributing pairs of universities as they were recruited using a random number generator. A priori randomization procedures specified that we assess for balance on the basis of students-to-therapist ratios at the counseling centers to ensure a balance in usual care across conditions. We used a biased coin technique^21^ in our randomization so that intervention and control universities would be approximately balanced on this ratio by the end of randomization.

### Measures

The primary outcome was reduction in overall ED psychopathology according to the Eating Disorder Examination-Questionnaire (EDE-Q)^22^ Global score (range, 0-6). Secondary outcome measures were abstinence from all ED behaviors (ie, binge eating, vomiting, laxative use, and excessive exercise) for the 4 weeks preceding assessment time points, assessed by the EDE-Q; ED behavior frequencies, including binge eating and compensatory behaviors (ie, vomiting, laxative use, and excessive exercise), assessed by the EDE-Q; depression as measured by the Patient Health Questionnaire-9^23^ (range, 0-27); anxiety as measured by the Patient-Reported Outcomes Measurement Information System anxiety short-form version 1.0 questionnaire^24^ (range, 4-20); ED-associated clinical impairment as measured by the Clinical Impairment Assessment^25^ (range, 0-48); academic impairment as measured by endorsement of the statements “withdrawn from a course due to academic difficulties caused by eating related issues” or “taken a leave of absence from a college due to eating related issues” at the postintervention and/or follow-up assessments since the last assessment was completed; and realized treatment access as defined by any use of the digital program in intervention and a positive response to the following question at the postintervention and/or follow-up assessments in the control group: “Have you had any treatment for eating related problems in the last [number adjusted based on time between assessments] months?” Engagement with the mobile intervention was defined as percentage of content completed.

### Statistical Analysis

Power analysis was based on the primary hypothesis testing (ie, determine effects of intervention vs control on EDE-Q Global) at the postintervention assessment. The power calculation took into account that cluster randomization occurred at the university level and the assumption that there would be approximately 25 students eligible per university. We assumed an intraclass correlation coefficient of 0.05 to 0.15. With little prior information on the effect size of our primary hypothesis, we assumed a medium effect size (Cohen *d*, 0.5) at the postintervention assessment. The estimated power ranged from 0.85 to 0.99 with 26 universities (13 universities per condition, 25 students per university) for a total sample of 650 students. We used multilevel mixed effects modeling to conduct the analysis of primary and secondary study aims including data from all participants in line with the intent-to-treat principle. Random effects were specified to account for the nested data structure of multiple assessments (level 1) within individual participants (level 2) and multiple individuals within each university (level 3). Fixed effects were specified to contrast (ie, to estimate change) the postintervention assessment with baseline and to contrast overall follow-up assessment (combining 1- and 2-year follow-up assessments) with baseline. Level 3 fixed effects were specified for the intervention effects (ie, intervention vs control) accounting for randomization at the university level. We specified a logit link for assessment of abstinence (binary outcome) and a log link for assessment of binge eating and compensatory behavior rate outcomes. For analyses of academic impairment, 2-sided Fisher exact tests were used to compare outcomes across conditions at each assessment (because of very small numbers of students reporting withdrawing from courses or taking leaves of absence), with statistical significance set at *P* < .05. Statistical analysis was performed with SPSS statistical software version 25 (IBM) and HLM7 software version 2013 (Scientific Software International, Inc). Data analysis was performed from February to September 2019.

## Results

### Descriptive Statistics

We randomized 28 US universities, with 1 university failing to recruit participants. For those that did recruit, the mean (SD) number of participants per university was 25.56 (21.91) participants, with a range of 2 to 88 participants. Six universities were from the East Coast, 4 from the South, 6 from the Midwest, 2 from the Intermountain West, and 9 from California.

**Figure 1.**
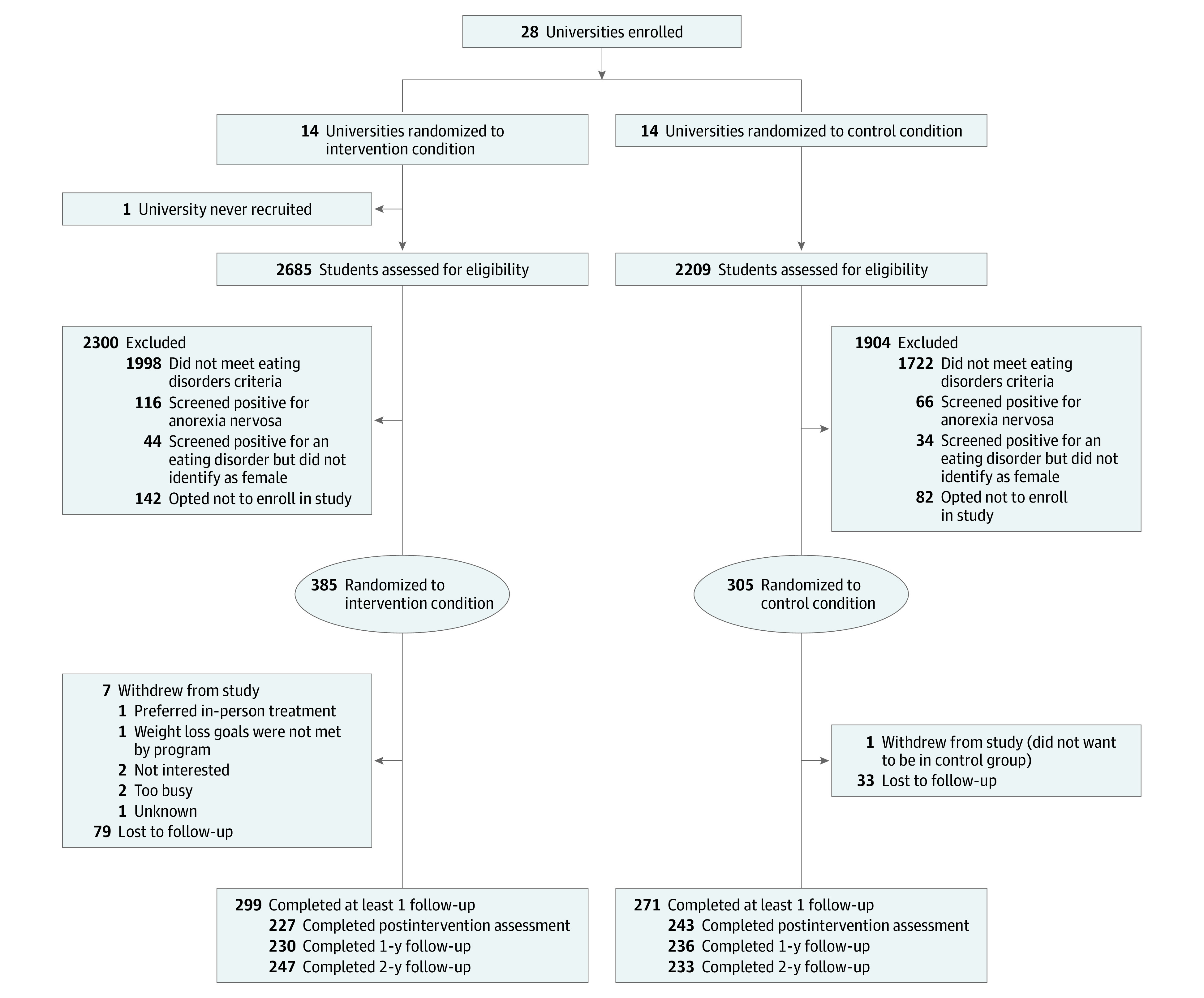
Participant Flow Diagram

Participants were recruited from January 12, 2014, to June 30, 2016, with data collection completed by November 30, 2018. We screened 4894 individuals and 914 were eligible. Of these, 690 (75.59%) agreed to participate (Figure 1); 385 were randomized at the university level to intervention and 305 to control. Follow-up rates can be seen in Figure 1, as well as reasons for withdrawal. Overall completion of at least 1 follow-up assessment was 82.6% (intervention, 299 of 385 participants [77.7%]; control, 271 of 305 participants [88.9%]).

The mean (SD) age of the 690 randomized participants was 22.12 (4.85) years. Most identified as White (414 participants [60.0%]), 118 (17.1%) identified as Asian or South Asian, 37 (5.4%) as Black or African American, 1 (0.1%) as Native Hawaiian or Pacific Islander, 3 (0.4%) as American Indian or Alaskan Native, 53 (7.7%) as multiracial, and 46 (6.7%) as other races. Regarding ethnicity, 120 (17.4%) identified as Hispanic. In terms of student status, 512 (74.2%) were undergraduate students, 171 (24.8%) were graduate students, 2 (0.3%) were postdoctoral fellows, and 4 (0.6%) were other. Mean (SD) body mass index (calculated as weight in kilograms divided by height in meters squared) was 25.69 (6.02). At entry, participants screened positive for *Diagnostic and Statistical Manual of Mental Disorders* (Fifth Edition) bulimia nervosa (137 participants [19.9%]), subthreshold bulimia nervosa (171 participants [24.8%]), binge-eating disorder (69 participants [10.0%]), subthreshold binge-eating disorder (66 participants [9.6%]), purging disorder (31 participants [4.5%]), or unspecified feeding or eating disorder (215 participants [31.2%]) (Table 1).

**Table 1.** Baseline Characteristics

Characteristic	Patients, No. (%)	
	Intervention (n = 385)	Control (n = 305)
Age, mean (SD), y	21.63 (4.19)	22.76 (5.52)
Race		
White	235 (61.0)	179 (58.7)
Asian or South Asian	78 (20.8)	40 (13.1)
Black or African American	17 (4.4)	20 (6.6)
Native Hawaiian or Pacific Islander	0	1 (0.3)
American Indian or Alaskan Native	1 (0.3)	2 (0.7)
Multiracial	27 (7.0)	26 (8.5)
Other	17 (4.4)	29 (9.5)
Hispanic ethnicity	55 (14.3)	65 (21.3)
Student status		
Undergraduate	290 (75.3)	222 (72.8)
Graduate	92 (23.9)	79 (25.9)
Postdoctoral fellow	1 (0.3)	1 (0.3)
Other	1 (0.3)	3 (1.0)
Body mass index, mean (SD)^a^	25.04 (5.53)	26.52 (6.49)
Diagnosis^b^		
Bulimia nervosa	87 (22.6)	50 (16.4)
Binge-eating disorder	41 (10.6)	28 (9.2)
Subthreshold		
Bulimia nervosa	89 (23.1)	82 (26.9)
Binge-eating disorder	28 (7.3)	38 (12.5)
Purging disorder	17 (4.4)	14 (4.6)
Unspecified feeding or eating disorder	122 (31.7)	93 (30.5)
Ever had an eating disorder	173 (44.9)	117 (38.4)
Treatment for an eating disorder in past year	61 (15.8)	36 (11.8)

^a^ Body mass index is calculated as weight in kilograms divided by height in meters squared.

^b^ Diagnosis is based on the Stanford-Washington University Eating Disorder Screen.

### Outcomes

Table 2 describes the outcome variables, and eTable 2 in Supplement 2 shows possible diagnoses for each assessment by condition. Table 3 summarizes the results of longitudinal mixed effects modeling of primary and secondary outcomes.

**Table 2.** Outcomes for Participants in the Intervention Condition Compared With the Control Condition

Variable	Condition, mean (SD)	
	Intervention (n = 385)	Control (n = 305)
Eating Disorder Examination-Questionnaire Global score		
Baseline	3.62 (1.13)	3.55 (1.07)
Postintervention	2.70 (1.33)	3.05 (1.22)
1 y	2.55 (1.32)	2.83 (1.27)
2 y	2.22 (1.32)	2.51 (1.32)
Abstinence from all eating disorder behaviors, % (No. of participants/total)^a^		
Baseline	2.11 (8/379)	2.30 (7/305)
Postintervention	8.81 (20/227)	6.61 (16/242)
1 y	14.89 (35/235)	10.46 (25/239)
2 y	19.28 (48/249)	15.61 (37/237)
Binge frequency, episodes in past 28 d, No.		
Baseline	9.19 (7.48)	9.34 (7.25)
Postintervention	4.53 (5.35)	5.41 (5.96)
1 y	4.16 (5.80)	4.68 (5.31)
2 y	3.11 (4.44)	4.28 (6.22)
Compensatory behavior frequency, episodes in past 28 d, No.^b^		
Baseline	10.11 (19.72)	8.53 (12.15)
Postintervention	4.22 (7.41)	5.18 (9.51)
1 y	4.10 (8.77)	4.30 (7.56)
2 y	3.20 (7.18)	3.35 (8.48)
Patient Health Questionnaire–9 score		
Baseline	11.09 (6.32)	11.08 (5.98)
Postintervention	8.21 (6.57)	9.40 (5.98)
1 y	7.77 (6.29)	8.72 (6.22)
2 y	7.32 (6.25)	8.36 (6.61)
Patient-Reported Outcomes Measurement Information System anxiety short-form score		
Baseline	11.26 (4.36)	11.09 (4.05)
Postintervention	9.61 (4.37)	10.14 (4.32)
1 y	9.10 (4.14)	9.61 (4.21)
2 y	8.84 (4.37)	9.41 (4.20)
Clinical Impairment Assessment score		
Baseline	25.52 (11.41)	24.60 (11.01)
Postintervention	19.95 (12.36)	20.98 (11.49)
1 y	17.82 (12.30)	19.65 (11.93)
2 y	15.66 (11.94)	17.29 (11.95)
Body mass index^c^		
Baseline	25.04 (5.53)	26.52 (6.49)
Postintervention	25.51 (6.23)	26.63 (6.70)
1 y	25.63 (6.36)	26.70 (6.82)
2 y	25.34 (6.21)	26.24 (6.46)
Course withdrawal due to eating disorder issues since last assessment, participants, No.		
Postintervention	8	7
1 y	8	6
2 y	7	5
Leave of absence due to eating disorder issues since last assessment, participants, No.		
Postintervention	4	4
1 y	6	1
2 y	3	3

^a^ Abstinence from all eating disorder behaviors involves abstinence from binge eating, vomiting, laxative use, and excessive exercise in the past 28 days.

^b^ Compensatory behavior frequency is the sum of compensatory behaviors in the past 28 days, including vomiting, laxative use, and excessive exercise.

^c^ Body mass index is calculated as weight in kilograms divided by height in meters squared.

**Table 3.** Estimated Effects of Intervention on Outcome Measures^a^

Outcome measures	Intervention effect, β (SE)		Intervention effect					
	Postintervention assessment	Follow-up	Postintervention assessment			Follow-up		
			*t*_1387_	*P* value	Effect size (*d*)	*t*_1387_	*P* value	Effect size (*d*)
Continuous measures								
Eating Disorder Examination-Questionnaire	−0.44 (0.10)	−0.39 (0.12)	−4.23	<.001	−0.40	−3.30	<.001	−0.35
Patient Health Questionnaire–9	−1.34 (0.53)	−1.28 (0.40)	−2.52	.01	−0.22	−3.18	.001	−0.21
Patient-Reported Outcomes Measurement Information System anxiety short-form	−0.65 (0.35)	−0.84 (0.32)	−1.86	.06	−0.15	−2.64	.008	−0.20
Clinical Impairment Assessment	−2.33 (0.94)	−3.19 (1.06)	−2.49	.01	−0.21	−3.01	.003	−0.28
Eating disorder behaviors, rate ratio (95% CI)^b^								
Abstinence (binary)	1.48 (0.48-4.62)	1.51 (0.63-3.58)	0.68^c^	.50		0.92^c^	.36	
Binge eating (rate)	0.82 (0.70-0.96)	0.81 (0.65-1.00)	−2.42^c^	.02		−1.94^c^	.05	
All compensatory behaviors (rate)^d^	0.68 (0.54-0.86)	0.76 (0.60-0.98)	−3.26^c^	<.001		−2.11^c^	.04	

^a^ A logit link was specified in the mixed effects model assessing effects on abstinence. A log link was specified in mixed effects models assessing effects on binge eating and compensatory behavior rates.

^b^ Eating disorder behaviors included binge eating episodes, or compensatory behaviors involving vomiting, laxatives, and/or excessive exercise in the past 28 days.

^c^ The *df* for these *t* statistics is 1392.

^d^ All compensatory behaviors is the sum of frequency counts of compensatory behaviors in the past 28 days, including vomiting, laxative use, and excessive exercise.

#### Primary Outcome

**Figure 2.**
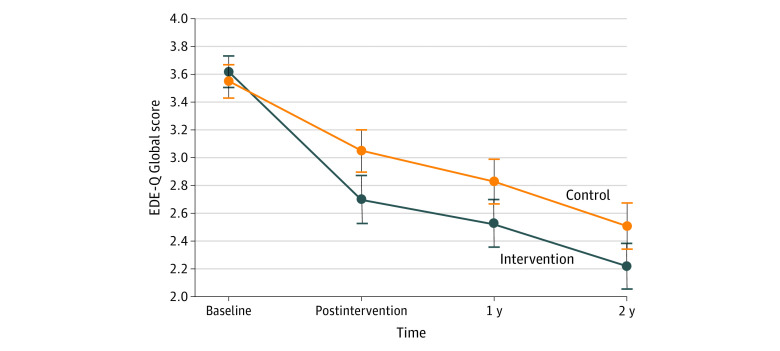
Observed Trajectories of the Eating Disorder Examination-Questionnaire (EDE-Q) Global Score Dots denote means and vertical lines and error bars denote 95% CIs.

There was a significantly greater reduction in EDE-Q Global score in the intervention group vs the control group at the postintervention assessment (β [SE], −0.44 [0.10]; *t*_1387_ = −4.23; *d* = −0.40; *P* < .001) and over follow-up (β [SE], −0.39 [0.12]; *t*_1387_ = −3.30; *d* = −0.35; *P* < .001). As shown in Figure 2, in the intervention group, the EDE-Q Global score decreased from 3.62 (95% CI, 3.51-3.73) at baseline to 2.70 (95% CI, 2.52-2.87) at the postintervention assessment. In the control group, the score decreased from 3.55 (95% CI, 3.43-3.67) at baseline to 3.05 (95% CI, 2.90-3.20) at the postintervention assessment. The median (interquartile range) EDE-Q Global score was 2.18 (1.09-3.06) for the intervention group at 2-year follow-up.

#### Secondary Outcomes

There was no significant difference between conditions in terms of abstinence from all ED behaviors at the postintervention assessment (odds ratio, 1.48; 95% CI, 0.48-4.62; *P* = .50) or at follow-up (odds ratio, 1.51; 95% CI, 0.63-3.58; *P* = .36). For binge eating frequency (ie, number of episodes in the last 28 days), there were significantly lower rates in the intervention group vs the control group at the postintervention assessment (mean [SD] for the intervention group, 9.19 [7.48] episodes at baseline and 4.53 [5.35] episodes at the postintervention assessment; rate ratio, 0.82; 95% CI, 0.70-0.96; *P* = .02) but not over follow-up (rate ratio, 0.81; 95% CI, 0.65-1.00; *P* = .05). For overall combined compensatory behavior frequencies, there were significantly lower rates in the intervention group vs the control group at the postintervention assessment (rate ratio, 0.68; 95% CI, 0.54-0.86; *P* < .001) and over follow-up (rate ratio, 0.76; 95% CI, 0.60-0.98; *P* = .04).

For depression, there was a significantly greater reduction in the intervention group vs the control group at the postintervention assessment (β [SE], −1.34 [0.53]; *t*_1387_ = −2.52; *d* = −0.22; *P* = .01) and over follow-up (β [SE], −1.28 [0.40]; *t*_1387_ = −3.18; *d* = −0.21; *P* = .001). There was no significant difference between conditions in terms of anxiety reduction at the postintervention assessment (β [SE], −0.65 [0.35]; *t*_1387_ = −1.86; *d* = −0.15; *P* = .06), but the difference over follow-up was significant (β [SE], −0.84 [0.32]; *t*_1387_ = −2.64; *d* = −0.20; *P* = .008). There was also a significantly greater reduction in ED-associated clinical impairment in the intervention group vs the control group at the postintervention assessment (β [SE], −2.33 [0.94]; *t*_1387_ = −2.49; *d* = −0.21; *P* = .01) and over follow-up (β [SE], −3.19 [1.06]; *t*_1387_ = −3.01; *d* = −0.28; *P* = .003). According to separate Fisher exact tests, no group differences emerged at any time point on withdrawing from a course (postintervention, *P* = .80; 1-year, *P* = .60; 2-year, *P* = .78) or taking a leave of absence (postintervention, *P* > .99; 1-year, *P* = .07; 2-year, *P* > .99) because of eating-related issues since the last assessment.

The majority of intervention participants (318 of 385 participants [83%]) began the intervention, whereas only 28% of control participants (76 of 271 participants with follow-up data available) reported obtaining ED treatment at any point. When compared this way, the odds of engagement with some form of ED-related intervention were more than 12 times greater for intervention vs control participants (odds ratio, 12.36; 95% CI, 8.73-17.51; *P* < .001). Regarding intervention engagement, among the 363 participants who created an account, participants completed a mean (SD) of 31% (37%) of the content offered. For year 1 participants, mean (SD) engagement was 17% (31%), and for years 2 and 3, it was 39% (38%). Among intervention participants, the percentage engagement was significantly associated with greater reduction in EDE-Q Global scores from baseline to the postintervention assessment (β [SE], −0.005 [0.001]; *t*_1385_ = −5.10; *P* < .001).

#### Subanalysis

Because the design of the intervention changed between year 1 (longer sessions, online only) and years 2 and 3 (shorter sessions, online, and mobile telephone app), the primary outcome analyses were rerun excluding year 1 participants. As in the full sample, there was a significantly greater reduction in EDE-Q Global scores in the intervention group vs the control group at the postintervention assessment (β [SE], −0.39 [0.12]; *t*_1376_ = −3.38; *d* = −0.37; *P* < .001) and over follow-up (β [SE], −0.38 [0.13]; *t*_1376_ = −2.89; *d* = −0.36; *P* = .004).

## Discussion

SB-ED, a digital CBT-guided self-help program for EDs, was associated with significantly greater reductions in the primary outcome, ED psychopathology, vs referral to usual care among college women at both postintervention assessment and over long-term follow-up. The controlled effect size at the postintervention assessment (*d* = 0.40) is in line with meta-analytic findings on the effect of in-person, therapist-led CBT vs inactive control on cognitive ED symptoms in patients with bulimia nervosa and binge-eating disorder (Hedge *g* = 0.24-0.34).^26^ The effect size is also similar to those from other randomized trials of digital interventions, both for EDs and other psychiatric conditions.^27,28^

Regarding secondary outcomes, although there were no differences in abstinence rates between conditions at any time point, the intervention demonstrated superiority in reducing ED psychopathology and all ED behaviors at postintervention and superiority in reducing ED psychopathology and compensatory behaviors over follow-up. Indeed, the median EDE-Q Global score (2.18) for the intervention group at 2-year follow-up was less than 2.3, which is the cutoff for a clinical disorder,^29^ suggesting half the sample no longer had a clinical ED, even if they were experiencing some behaviors. Furthermore, compared with control, there was a large, significant reduction in binge frequency in the intervention group from a mean (SD) of 9.19 (7.48) episodes at baseline to 4.53 (5.35) episodes at the postintervention assessment, but the results were not significant at follow-up because both groups demonstrated large reductions. The intervention was also associated with significant reductions in depression and ED-associated clinical impairment at the postintervention assessment and over follow-up compared with control, as well as with significant reductions in anxiety over follow-up. It is important to note that body mass indexes remained constant even as ED attitudes and pathology improved, demonstrating that these changes were not associated with changes in weight. Furthermore, although academic impairment outcomes did not differ between groups, the rates of course withdrawal and taking a leave of absence were quite low overall. Finally, with regard to realized treatment access, the intervention was far superior: 83% of students offered the intervention began it, whereas only 28% of students in the control group reported seeking treatment for their ED at any point over the 2-year follow-up. Overall, the intervention was associated with significantly greater improvements than control in the primary outcome, ED psychopathology, as well as in binge eating, compensatory behaviors, depression, and clinical impairment at postintervention assessment, with these gains sustained through longer-term follow-up for all outcomes except binge eating. These differences are notable given the control group demonstrated substantial improvement.

### Strengths and Limitations

Strengths of this study include the large number of participants (a particular challenge in intervention trials for EDs), diversity of the population, broad inclusion criteria, delivery on a national scale, and long-term follow-up. In addition, we optimized the service over time, an important innovation for implementing psychosocial and digital interventions.^19,30^

Regarding limitations, first, overall engagement with the intervention was 31%. Yet, even with this level of engagement, the intervention group demonstrated significant improvement vs the control group. Furthermore, this level of engagement is consistent with engagement with mental health apps in the real world; one review^31^ indicated that median retention rates for mental health apps were 3.9% for 15 days and 3.3% for 30 days. Future research should address the issue of improving engagement with mental health apps, including SB-ED. Second, the population was recruited though an online screen; thus, ED status was determined on the basis of self-report rather than diagnostic interview. Importantly, however, this approach is consistent with what would be required to sustainably implement digital screening and intervention broadly on university campuses.^5^ In addition, current results suggest the superiority of the intervention vs referral to usual care, but future research may wish to compare to other control conditions (eg, in-person CBT).

## Conclusions

Overall, these findings support the use of a coached, digital CBT intervention, compared with referral to usual care, for college women with a wide range of EDs. Given its scalability, a digital CBT-guided self-help intervention for EDs has great potential to address the wide treatment gap for this problem.

## Notes

Supplement 1. **Trial Protocol**

Supplement 2. **eTable 1.** Outline of the Final Version of the *Student Bodies-Eating Disorders* Mobile Intervention **eTable 2. **Frequencies of Possible Diagnoses at Post-intervention and Follow-up Time Points

Supplement 3. **Data Sharing Statement**
