# Supplementary material for: Effectiveness of a Digital Cognitive Behavior Therapy–Guided Self-Help Intervention for Eating Disorders in College Women: A Cluster Randomized Clinical Trial
Source: JAMA Netw Open. 2020 Aug 31;3(8):e2015633. doi: 10.1001/jamanetworkopen.2020.15633 (PMC7489868; doi:10.1001/jamanetworkopen.2020.15633)
Supplement: Supplement 1. — Trial Protocol [file jamanetwopen-e2015633-s001.pdf]

eMethods. Institutional Review Board study protocol with amendments

Project Name: Using Technology to Improve Eating Disorders Treatment

Principal Investigators (PIs): Denise Wilfley Ph.D. & C. Barr Taylor, M.D.

1. **Background/Rationale/Literature**

   Despite the pressing need for intervention, current treatment delivery standards for eating disorders (EDs) on college campuses are hindered by minimal screening efforts, limited access to evidence based care, and exceeded capacity for intervention.[1] College campuses are faced with an elevated prevalence of EDs as the risk for onset is highest in late adolescence and early adulthood.[2] Rates of EDs are estimated as high as 13.5% and 3.6% for US college age women and men, respectively.[3] However, less than 20% of students who screen positive for EDs report receiving treatment.[3] Delays in treatment delivery result in prolonged illness, disease progression, poorer prognosis, and greater likelihood of relapse.[4] Reported barriers to service utilization on college campuses include unawareness of services, skepticism of treatment effectiveness, and lack of perceived need.[5] Online screening and intervention offers an ideal medium through which to increase access to care, improve outcomes, reduce costs, and establish a self-sustaining system for providing mental health services.[6-8] Online interventions have been successfully used for treatment and prevention of EDs,[9-16] with high user acceptability given their accessible and anonymous format. However, an online platform through which screening and tailored interventions are provided to individuals with various levels of risk or ED diagnosis has not been deployed. Our research team has successfully developed a comprehensive, internet-based platform through which we identify and offer tailored evidence-based interventions to individuals across the ED risk and diagnostic spectrum using minimal person based resources. The newest intervention in our suite of programs has not yet been tested in a large scale trial or via platform delivery. The aim of this study is to conduct the first national deployment of our comprehensive platform and demonstrate that our transdiagnostic guided self-help program, Student Bodies-Eating Disorders (SB-ED), improves access, costs, and outcomes of mental health care for EDs.

2. **Hypothesis/Key Questions**

   We believe our platform will demonstrate a significant impact on access, costs, and outcomes for the treatment of EDs. Integrated screening and intervention will result in increased access (i.e., receipt of ED treatment) to mental health care by identifying students with an ED, automatically directing them to easily accessible care, and tracking their progress over time. Comparison of implementation and service costs between SB-ED and usual care has the potential to demonstrate improved costs (i.e., positive net benefit from a health care payor's perspective) for the treatment of EDs. Our previous small scale efficacy testing suggests that SB-ED will be associated with improved outcomes (i.e., decrease in ED pathology and functional impairment) over referral to usual care.

3. **Research Objectives and Purpose**

   Our research goal is to demonstrate that delivery of SB-ED via our online platform significantly improves the system of care for EDs on college campuses. The goal of SB-ED is to target the core pathology of ED diagnosis and to provide tailored content to address specific ED features based on individuals' symptom profile. Though our target conditions are classified as categorical diagnoses, the shared latent pathology of EDs across the binge/purge spectrum makes a comprehensive, diagnostic approach ideal for treatment.[17,18] Our approach is comparable to the transdiagnostic model that has been applied to the treatment of EDs,[19,20] which addresses pathology common across ED diagnoses but incorporates tailored intervention components specific to the presenting symptoms. Our interventions include mobile technology that allows users to provide ongoing, real-time assessment of symptom progression. We also use clinical data management "dashboards," sophisticated online tools that allow moderators to visually represent individual users' progress over the course of the intervention to efficiently monitor multiple users at one time. These tools set the stage for scale up by increasing relevance and efficiency.

4. **Research Methods**

   Our approach will use an evidence based stepped care framework.[21-24] Given that the need for treatment outweighs clinician capacity,[6] guided self-help interventions are recommended as a first line treatment,

with standard in person treatment offered in the event of worsening or maintenance of symptoms. Flexible online modalities allow us to expand this paradigm by tailoring interventions over the course of treatment, making the intervention more interactive than guided self-help books and, by offering interventions via computer and phone, more congruent with students' routines. Consistent with the guided self-help model, moderators will coach and motivate students through the program. The program itself will include twenty eight colleges, each of which will be allocated at random to receive either SB-ED or referral to usual care (i.e., treatment per protocol at students' corresponding college's mental health services center). Campus selection criteria are based on interest and population size. We will recruit 28 schools across the United States. We will require that colleges identify a clinical liaison in the student mental health services center. We will seek IRB approval or acknowledgment at all participating schools and obtain informed consent from student participants. The clinical liaisons will partner with moderators at study sites to deploy the intervention, field clinical referrals, and communicate with study staff regarding any adverse events. The clinical liaison will also update the PIs regarding campus staff and students' impressions of the initiative. As another incentive, schools will receive the evidence based Student Bodies-Targeted and StayingFit programs to offer students who do not have an ED, regardless of study condition. This will complement the screening efforts and afford schools a comprehensive solution to improve the health and well-being of their students. Students will complete an online screen and then be triaged to appropriate care based on their presenting symptoms: Student Bodies Eating Disorders, Student Bodies Targeted, Staying Fit, or a clinical referral. We will enroll at least 650 students who screen positive for a DSM 5 clinical or subclinical ED, defined as DSM 5[25] bulimia nervosa (BN), binge eating disorder (BED), subclinical BN (sub BN), subclinical BED (sub BED), or purging disorder (PD). Individuals who screen positive for DSM 5 anorexia nervosa (AN) will be offered a referral, given that the medical severity of AN warrants more intensive clinical intervention. All participants will take the online screen which assesses demographics (sex, race/ethnicity, age), ED related attitudes and behaviors, and clinical impairment. Self-reported height and weight will also be collected to calculate BMI. Participant outcomes will be assessed with the Eating Disorder Examination Questionnaire (EDE-Q) version 6,[26] a 39 item measure of eating pathology and symptom severity. The Weight Concerns Scale (WCS) is a reliable and valid measure of eating attitudes and predicts ED risk.[16,27-29] Measures of comorbidities include the Patient Health Questionnaire (PHQ-9),[30] a measure of depression symptom severity; the Patient-Reported Outcomes Measurement Information System (PROMIS) anxiety short-form v. 1.0-Anxiety4a questionnaire,[31] a measure of anxiety severity; and a measure of alcohol use.[32] Measures of impairment include the Clinical Impairment Assessment (CIA),[33,34] a 16-item measure of clinical impairment due to ED-related problems; and the College Student Eating Disorder Related Academic Impairment Scale (CS-ED AIS), an 8-item self-report measure created by the research team to measure ED related impairment across academic domains. Additionally, we will capture continuous measures of symptoms across a range of mental health problems relevant to college students via administration of the Counseling Center Assessment of Psychological Symptoms 34 (CCAPS-34).[6] The CCAPS-34 is a 34 item scale condensed from the CCAPS-62[7] and can be completed on average in 2-3 minutes.[6] We are partnering with a software platform (Lantern) to provide online, evidence based interventions to defined populations. Specifically, our platform includes StayingFit (for low-risk students), Student Bodies-Targeted (for high-risk students) and the program that will be examined in this study, SB ED. All programs are derived from evidence based, cognitive behavioral interventions.[16,35] (Note, in the current study, the only intervention under evaluation will be SB-ED.) The platform is structured so that programs can be revised and enhanced depending on outcome and can address multiple mental health issues. SB-ED is the latest program in a set of programs we have developed. The intervention is a structured, cognitive behavioral program, derived from manual-based cognitive behavioral therapy. The intervention targets the core ED pathology (e.g., extreme dietary restraint, overvaluation of shape and weight, binge eating, compensatory behaviors), focusing on helping users develop regular eating patterns, self-control strategies, problem solving skills, and relapse prevention tools for maintenance of behavior change. SB-ED also provides tailored content to address specific ED features based on individuals' symptom profile. We include content related to coping with negative emotions including skills training and inclusion of elements from dialectic behavior therapy (e.g., acceptance of negative emotions, mindfulness, and mindful eating), as well as psychoeducational content on social skills and cognitive restructuring. The program includes symptom checklists (assessing weight, number of meals per day, and number of binges and compensatory behaviors per day), journal exercises and activities, and a discussion group (through which users can interact anonymously to discuss reactions to the program, seek advice, or ask questions to peers or the moderator).[36] Program moderators will be clinical psychologists or advanced graduate student

trainees under the supervision of a clinical psychologist or psychiatrist. Campus clinical liaisons may also moderate the program after receiving training. Moderators will guide and motivate students in using the program, check daily to monitor participant progress and provide individualized feedback, and moderate the discussion group. Senior personnel will provide additional oversight by reviewing weekly behavior logs and discussion group postings. SB-ED includes exciting, new technological features to make the platform more attractive and efficient, to increase engagement and monitor individuals' progress in real time.

4.1 Amendments to the Research Methods

The initial plan was to enroll and recruit from 28 U.S. universities. The study team enrolled and randomized 30 universities, however 3 dropped out before recruitment at those sites began. As such, 27 universities participated in the data collection. (Approved by IRB on 4/02/14)

The program also started with the use of a discussion board, to allow users to interact with one another. However, as the program platform changed, the discussion board functionality was removed. A separate discussion board was created outside of the platform for users to engage with if they wished. To supplement this interaction with moderators, users of the SB-ED program were assigned a coach, who was a clinical psychologist or advanced graduate student trainees under the supervision of a clinical psychologist or psychiatrist. Coaches could message back and forth through a secure system with the users, and help them understand the program content and apply it to their own situations. The coaches also helped to increase engagement by reaching out to the users and reminding them about their next session. (Approved by IRB on 07/06/2015).

The SB-ED program was changed to improve engagement from an 9 unit weekly program to a 40 session daily program. The content from the nine sessions was primarily the same, but spread out across more, shorter, sessions. (Approved by IRB on 07/06/2015).

5. Study Participants

We will use recruitment methods based on our previous trials. Potential participants will be recruited from flyers posted at local academic institutions, campus mailings and emails, and mass media. Schools may also elect to deliver the screen in a population based format to all or a defined subset of students (e.g., mandated screens; including the screen with new student orientation materials; offering it at routine medical visits). Members from the research team will assist colleges to advertise the screen and/or assist school administrators in implementation of a population based delivery.

Participant recruitment will occur in three waves, of three consecutive academic calendar years. We will terminate recruitment from February through the end of the academic year, as students requiring clinical referral will not have adequate time to pursue a standard treatment regimen (12 weekly sessions) at the mental health services center before the end of the semester. Entry into discussion groups will be rolling, as in group psychotherapy in clinical settings. Given that all assessments will be completed online, follow up will occur throughout the year, regardless of schools' schedules. Online gift cards will be used to incentivize participants to complete follow-up assessments.

Given that rates of EDs in males are much lower than in females, our study enrollment goals will be limited to females; however, males with EDs will be welcome to participate. The interventions will be provided to males in sex specific groups. Data collected from men will be exploratory.

Participants will include students, ages 18 to 30, at the participating colleges and universities who screen positive for a DSM 5 clinical or subclinical ED (excluding AN). We intend to enroll 650 students into the trial, equivalent to 25 students per site. Eligible participants will not be currently undergoing treatment for an ED, have access to the internet, and not be acutely suicidal.

Interested individuals at all of the schools, regardless of the randomization condition, will be eligible to complete the online screen. The screen takes less than 5 minutes to complete and can be administered online via computer or Smartphone. Students immediately receive screen results in an email, indicating whether an intervention or clinical referral is recommended based on their presenting risk or symptom status. Individuals screening positive for a clinical or subclinical ED (excluding AN) will be invited to

participate in the trial. A study description will be provided in the email with screen results. Following the study description, consent will be obtained in a secure online format as well. For those prompted to an intervention, a link to the program, program description, a randomly generated username, and a randomly generated password are also included in this email. Students are encouraged to log in to the intervention, change their username and password to an anonymous one of their preference, and begin the program. Students who warrant a clinical referral (either because they are in the control condition or because they screen positive for AN) will receive an immediate email as well indicating this recommendation, with contact information for the student mental health services center. All students will receive an automatic email prompt one week following screen completion, reminding them of their screen feedback and encouraging them to pursue the recommended strategy. Preferences for additional follow up procedures can be specified by the schools on a campus by campus basis.

5.1 Study Participant Amendments

Additional recruitment methods were used besides the ones originally detailed. For example, the study team conducted tabling events on campuses to screen students, as well as presentations in classes at student group meetings. (Approved by IRB on 02/19/15)

Participant recruitment was originally delayed to January of 2014, due to a delayed launch of the program on campuses. It was determined that campus mental health services would connect students with therapeutic options to bridge the summer if the students received a referral close to the end of their spring semesters. As such, recruitment was continuous from the start of January 2014 until July of 2016. (No IRB approval given for this change, as recruitment was approved for anytime following initial approval on 09/05/13).

Regarding eligibility criteria, participants were allowed to enroll as long as they were 18 years old or over, and students at the university participating in the study. It was decided that participants could also be receiving in-person treatment (an original exclusion criteria), because referral to in-person care was offered to those who did not progress by mid-program. (Approved by IRB on 05/19/14)

The screen result was still immediate, but did not arrive via email (as previously described). The screen result was presented at the end of their survey. For the participants in the referral condition, they immediately received contact information for their campuses resources. For those that were in the intervention condition, they were given a link to complete their first baseline, should they choose to participate. The participants were able to progress from the screen, to the baseline, to the program login page, and were allowed to create their own username and password to being the program. (Approved by IRB on 11/05/14)

6. Statistical Analysis

Stanford University will serve as the Data Coordinating Center (DCC). Drs. Trockel and Jo will be responsible for maintaining the dataset and ensuring the data remain blind to the PIs. Data will be stored on a HIPAA compliant server on a Power Mac GS with secure access available to Drs. Trockel and Jo. Statistical analysis will utilize SPSS 19.0 and Mplus, updated yearly, as well as other specialized statistical programs when necessary.

Primary Analysis for Specific Aim 1. The primary outcome of this project is eating disorder (ED) symptom severity, as assessed by the Eating Disorder Examination Questionnaire (EDE-Q). We will compare EDE-Q total scores across the two intervention arms (SB-ED versus usual care).

Secondary Aims 1, 2, & 3. For Secondary Aim 1, the effect of SB-ED, compared to usual care, on realized treatment access will be estimated by self-report of receiving ED treatment between baseline and 6-month follow up. In Secondary Aim 2, we will compare ED behavior (i.e., reported binge and purge behaviors) abstinence rates between the two arms, as assessed by the EDE-Q. For Secondary Aim 3, we focus on continuous measures of symptom severity for depression, anxiety, ED associated clinical impairment, and ED associated academic impairment. Secondary Aim 4. We will conduct the economic evaluation analysis from the payor's perspective, meaning the paid charges for providers.

The outcome of interest is the net benefit of treatment, comparing SB-ED to referral to usual care at 24 months. We define net benefit as the difference between cost of providing care (from the payor perspective) and cost of SB-ED. Secondary Aim 5. For our primary outcome, EDE Q total scores, we will examine baseline variables (e.g., site and patient characteristics, patient comfort with and acceptance of technology based intervention) as potential moderators. Potential mediators of subsequent outcomes include baseline to 6 month reduction in weight and shape concerns and dietary restraint. Similar potential mediators and moderators will be explored for our secondary outcome pertaining to abstinence rates. For our secondary outcome pertaining to treatment access, potential moderators consist of variables such as patient characteristics and site characteristics. In addition to the above, we will examine within group SB-ED group correlations between treatment adherence variables (e.g. completion of self-monitoring forms) and changes in EDE-Q total scores.

7. Potential Problems

<u>Risks to the Participants</u>

There are no high risk or hazardous aspects of our proposed intervention. One potential risk includes accidental disclosure of private, identifiable information. The information will be housed on a HIPAA protected server and will only be downloaded from the server after being de-identified. We recognize that a program to provide guided self-help for eating disorders may promote increased focus on body image and eating attitudes and behaviors, more than that which may exist before starting the program. Due to increased sensitivity, the participants in the program will be monitored by trained moderators, supervised by licensed psychologists, at Stanford University, Washington University, or the participating study sites. Given our use of a stepped care approach, additional risks may present themselves. This study involves individuals who are screening positive for eating disorders; individuals in the intervention condition are being offered an online moderated guided self-help intervention. However, referral to in person treatment will be recommended to users in the event of worsening symptoms. It is possible that without intensive care, participants' symptoms could remain stagnant or increase over the course of the intervention. If any participant appears to be in crisis or exhibiting symptom worsening, appropriate action will be taken based on established suicide and crisis assessment protocol.

<u>Minimization of Risk</u>

The overall risks for this study are minimal. Participants' diagnostic information, obtained from the screen, will be kept private to minimize potential embarrassment related to diagnosis. There are minimal risks for participating in the program. Students will be provided with the contact information of their program moderator, study staff, and students' corresponding college's clinical liaison at the mental health services center. All information will be kept on a HIPAA compliant server. Prior to implementation, the server will be audited by a third party to ensure all technical and protocol requirements are met. The technology meets current standards for privacy; subsequent versions of iPhone technology that are released that have the same security protections may be used, as they remain in compliance with the current privacy standards.

Moderators will monitor students' progress via the online intervention clinical data management "dashboard" and online group discussion board. The dashboard is a sophisticated online tool that allows clinicians to visualize individual users' progress to efficiently monitor multiple users at one time. Moderators will be prompted to send email message reminders to participants to complete the logs. To ensure HIPAA compliance, email messages will be standardized messages merely alerting users that they have a message on their program homepage or a reminder to complete their behavior logs online.

The discussion board provides an anonymous arena for multiple users to post comments, offer feedback, and give reactions to the program content. As such, this may give participants a space to post demeaning or inappropriate comments to other participants. We have a zero tolerance policy for such postings and we will caution against them in three ways: (1) participating students can flag negative posts (four flags will result in removal from the program); (2) moderators will conduct random, daily site checks; and (3) the network will track negative key words and alert study staff to intervene through the clinical dashboard. As an extra precaution, we will address these issues in the subjects' online "netiquette" (i.e., internet etiquette) training prior to starting the program. It is worthwhile to note that we have used these practices successfully in studies involving over 1000 students participating in over 30 online groups, and we have never received complaints or had adverse events as a result of the online program or discussion board.

Each week, the progress, process, and postings in the electronic discussion group and behavior logs will be reviewed by a psychologist or psychiatrist through the Dashboard. If students do not achieve 50% symptom reduction by session 6 of the intervention or symptom abstinence following the intervention, they will be offered an on campus referral. At the 6-month assessment, students who remain in the sub-clinical or clinical eating disorder range will be referred to the student mental health service center.

The senior behavioral scientist will train program moderators, including how to address safety issues. Materials will also be provided to the college's clinical liaison at the mental health services center regarding safety issues. In addition, the clinical dashboard and discussion board will be accessed by the study moderator at least once daily. Discussion board postings will be monitored for signs of dangerous changes in weight related behaviors (i.e., bingeing, purging, or dietary restriction), severe depression, possible suicide/self-injury, or harm to others. Students who do not report 50% or greater symptom reduction by session 6 of the intervention or symptom abstinence following the intervention will be offered an on campus referral. If any participant appears to be in crisis, appropriate action will be taken based on established suicide and crisis assessment protocol (see below). Any adverse event will be reported promptly to NIMH and to the IRBs at the participating institutions within 72 hours by email.

Crisis Protocol

Potential Risks

There are no high risk or hazardous aspects of our proposed program; however, we have established a safety protocol should we feel participant safety is compromised.

School clinical liaisons will be notified, as appropriate, should there be harmful postings on our discussion board or drastic increases or decreases in students' weights. Also, we recognize that the program may promote increased focus on body image and eating habits, more than that which may exist before starting the program. Awareness of and sensitivity for this issue will be covered in moderator training and study moderators will remain attuned to website postings that may indicate harmful reactions. The discussion board provides an arena for posting, which may give participants a space to post demeaning or inappropriate comments to other participants. We have a zero tolerance policy for such postings and we will caution against them in three ways: (1) participating students can flag negative posts (four flags will result in removal from the program); (2) moderators will conduct random, daily site checks; and (3) the network will track negative key words and alert study staff to intervene. It is worthwhile noting that while all of these issues are "potential risk" our recently completed trial, involving 480 women for up to 3 years, did not have any complaints or adverse events as a result of the online program or discussion board.

Potential Risk Amendment

As noted in the procedures section, a coach messaging system replaced the primary function of the discussion board. As such, the same detailed protocol for ensuring the safety of the participants on the discussion board was applied to the coach messages.

Adequacy of Protection against Risk

Protections Against Risk

The overall risks for this study are minimal. No identifying information will be kept with the self-report questionnaires, or discussion boards. There are minimal risks for participating in the program. Some participants might make inappropriate comments, report inaccurate information or in other ways "misuse" the chat groups. To minimize these issues, all groups will be moderated by a trained research team member. Within the program, there will also be a discussion of “netiquette” to encourage appropriate and supportive online postings and minimize stigmatization. Students will be provided with contact information for appropriate campus resources.

The Discussion Board will be accessed by the study moderator at least once daily. Discussion Board postings will be monitored for signs of dangerous weight related behaviors, severe depression, possible suicide/self-injury, or harm to others. Any student who reports the above behaviors or a BMI that is either above the 95th percentile or below the 5th percentile will be given an automatic referral to a physician. School administrators will be notified as appropriate.

Protection Against Risk Amendment

As noted in the procedures section, a coach messaging system replaced the primary function of the discussion board. As such, the same detailed protocol for ensuring the safety of the participants on the discussion board was applied to the coach messages.

REFERENCES

1. Grace TW. Health problems of college students. *J Am Coll Health.* 1997 May;45(6):243-250.
2. Smink FR, van Hoeken D, Hoek HW. Epidemiology of Eating Disorders: Incidence, Prevalence and Mortality Rates. *Curr Psychiatry Rep.* 2012 May .
3. Eisenberg D, Nicklett EJ, Roeder K, Kirz NE. Eating disorder symptoms among college students: prevalence, persistence, correlates, and treatment-seeking. *J Am Coll Health.* 2011;59(8):700-707.
4. American Psychiatric Association. Treatment of patients with eating disorders, third edition. American Psychiatric Association. *Am J Psychiatry.* 2006 Jul;163(7 Suppl):4-54.
5. Eisenberg D, Hunt J, Speer N, Zivin K. Mental health service utilization among college students in the United States. *J Nerv Ment Dis.* 2011 May ;199(5):301-308.
6. Kazdin AE, Blase SL. Rebooting Psychotherapy Research and Practice to Reduce the Burden of Mental Illness. *Perspect Psychol Sci.* 2011;6(1):21-37.
7. Andersson G. Using the Internet to provide cognitive behaviour therapy. *Behav Res Ther.* 2009 Mar;47(3):175-180.
8. Hollon SD, Munoz RF, Barlow DH, et al. Psychosocial intervention development for the prevention and treatment of depression: promoting innovation and increasing access. *Biol Psychiatry.* 2002 Sep;52(6):610-630.
9. Carrard I, Crepin C, Rouget P, Lam T, Golay A, Van der Linden M. Randomised controlled trial of a guided self-help treatment on the Internet for binge eating disorder. *Behav Res Ther.* 2011 Aug;49(8):482-491.
10. Carrard I, Fernandez-Aranda F, Lam T, et al. Evaluation of a guided internet self-treatment programme for bulimia nervosa in several European countries. *Eur Eat Disord Rev.* 2010 Sep .
11. Jacobi C, Volker U, Trockel MT, Taylor CB. Effects of an Internet-based intervention for subthreshold eating disorders: a randomized controlled trial. *Behav Res Ther.* 2012 Feb;50(2):93-99.
12. Lindenberg K, Moessner M, Harney J, McLaughlin O, Bauer S. E-health for individualized prevention of eating disorders. *Clin Pract Epidemiol Ment Health.* 2011;7:74-83.
13. Ljotsson B, Lundin C, Mitsell K, Carlbring P, Ramklint M, Ghaderi A. Remote treatment of bulimia nervosa and binge eating disorder: a randomized trial of Internet-assisted cognitive behavioural therapy. *Behav Res Ther.* 2007 Apr;45(4):649-661.
14. Pretorius N, Arcelus J, Beecham J, et al. Cognitive-behavioural therapy for adolescents with bulimic symptomatology: the acceptability and effectiveness of internet-based delivery. *Behav Res Ther.* 2009 Sep;47(9):729-736.
15. Sánchez-Ortiz VC, Munro C, Stahl D, et al. A randomized controlled trial of internet-based cognitive- behavioural therapy for bulimia nervosa or related disorders in a student population. *Psychological Medicine.* 2010;41(02):407-417.
16. Taylor CB, Bryson S, Luce KH, et al. Prevention of eating disorders in at-risk college-age women. *Arch Gen Psychiatry.* 2006 Aug;63(8):881-888.
17. Insel T, Cuthbert B, Garvey M, et al. Research domain criteria (RDoC): toward a new classification framework for research on mental disorders. *Am J Psychiatry.* 2010 Jul(7):748-751.
18. Keel PK, Brown TA, Holland LA, Bodell LP. Empirical classification of eating disorders. *Annu Rev Clin Psychol.* 2012;8:381-404.
19. Fairburn CG, Cooper Z, Doll HA, et al. Transdiagnostic cognitive-behavioral therapy for patients with eating disorders: a two-site trial with 60-week follow-up. *Am J Psychiatry.* 2009 Ma;166(3):311-319.
20. Fairburn CG, Cooper Z, Shafran R. Cognitive behavior therapy for eating disorders: a "transdiagnostic" theory and treatment. *Behav Res Ther.* 2003 May;41(5):509-528.
21. Mitchell JE, Agras S, Crow S, et al. Stepped care and cognitive-behavioural therapy for bulimia nervosa: randomised trial. *Brit J Psychiat.* 2011;198(5):391-397.
22. Wilson GT, Zandberg LJ. Cognitive–behavioral guided self-help for eating disorders: Effectiveness and scalability. *Clin Psychol Rev.* 2012;32(4):343-357.
23. Striegel-Moore RH, Wilson GT, DeBar L, et al. Cognitive behavioral guided self-help for the treatment of recurrent binge eating. *J Consult Clin Psychol.* 2010 Jun;78(3):312-321.
24. Wilson GT, Wilfley DE, Agras WS, Bryson SW. Psychological treatments of binge eating disorder. *Arch Gen Psychiatry.* 2010 Jan;67(1):94-101

25. American Psychiatric Association. DSM-5 Proposed Diagnostic Criteria for Feeding and Eating Disorders. 2012; http://www.dsm5.org/proposedrevision/Pages/FeedingandEatingDisorders.aspx. Accessed July 2, 2011.
26. Fairburn CG, Beglin S. Eating Disorder Examination Questionnaire (EDE-Q 6.0). In: Fairburn CG, ed. *Cognitive Behavior Therapy and Eating Disorders*. New York: Guilford Press; 2008.
27. Jacobi C, Abascal L, Taylor CB. Screening for eating disorders and high-risk behavior: Caution. *Int J Eat Disord.* 2004 Nov;36(3):280-295.
28. Killen JD, Taylor CB, Hayward C, et al. Pursuit of thinness and onset of eating disorder symptoms in a community sample of adolescent girls: a three-year prospective analysis. *Int J Eat Disord.* 1994 Nov;16(3):227-238.
29. Killen JD, Taylor CB, Hayward C, et al. Weight concerns influence the development of eating disorders: a 4-year prospective study. *J Consult Clin Psychol.* 1996 Oct;64(5):936-940.
30. Kroenke K, Spitzer RL, Williams JB. The PHQ-9: validity of a brief depression severity measure. *J Gen Intern Med.* 2001 Sep;16(9):606-613.
31. Pilkonis PA, Choi SW, Reise SR, et al. Item banks for measuring emotional distress from the patient-reported outcomes measurement information system (PROMIS): Depression, anxiety, and anger. *Assessment*. 2011;18(3):263-283.
32. Khaylis A, Trockel M, Taylor CB. Binge drinking in women at risk for developing eating disorders. *Int J Eat Disord.* 2009 Jul;42(5):409-414.
33. Vannucci A, Kass AE, Sinton MM, et al. An examination of the Clinical Impairment Assessment among women at high risk for eating disorder onset. *Behav Res Ther.* 2012 Jun;50(6):407-414.
34. Bohn K, Doll HA, Cooper Z, O'Connor M, Palmer RL, Fairburn CG. The measurement of impairment due to eating disorder psychopathology. *Behav Res Ther.* 2008 Oct;46(10):1105-1110.
35. Sinton MM, Taylor CB. Prevention: Current Status and Underlying Theory. In: Agras WS, ed. *Oxford Handbook of Eating Disorders*. New York: Oxford Press; 2010:307-331.
36. Kass AE, Trockel MT, Safer DL, et al. Internet-based preventive intervention for reducing eating disorder risk: A randomized controlled trial comparing guided with unguided self-help. *Behav Res Ther.* 2014 Dec;63:90-98.
