# Supplementary material for: Effectiveness of a Digital Cognitive Behavior Therapy–Guided Self-Help Intervention for Eating Disorders in College Women: A Cluster Randomized Clinical Trial
Source: JAMA Netw Open. 2020 Aug 31;3(8):e2015633. doi: 10.1001/jamanetworkopen.2020.15633 (PMC7489868; doi:10.1001/jamanetworkopen.2020.15633)
Supplement: Supplement 2. — eTable 1. Outline of the Final Version of the Student Bodies-Eating Disorders Mobile Intervention eTable 2. Frequencies of Possible Diagnoses at Post-intervention and Follow-up Time Points [file jamanetwopen-e2015633-s002.pdf]

Supplementary Online Content

Fitzsimmons-Craft EE, Taylor CB, Graham AK, et al. Effectiveness of a digital cognitive behavior therapy–guided self-help intervention for eating disorders in college women: a cluster randomized clinical trial. *JAMA Netw Open*. 2020;3(8):e2015633. doi:10.1001/jamanetworkopen.2020.15633

eTable 1. Outline of the Final Version of the *Student Bodies-Eating Disorders* Mobile Intervention
eTable 2. Frequencies of Possible Diagnoses at Post-intervention and Follow-up Time Points

This supplementary material has been provided by the authors to give readers additional information about their work.

© 2020 Fitzsimmons-Craft EE et al. *JAMA Network Open.*

eTable 1. Outline of the Final Version of the *Student Bodies-Eating Disorders* Mobile Intervention

Unit	Session Numbers	Unit Description
**Introduction**	1-2	This unit orients the user to the program and provides essential information about eating disorders, body image, and cognitive-behavioral therapy (CBT). Self-monitoring of meals is introduced to help the user keep track of eating patterns.
**Reducing Disordered Eating Behaviors**	3-10	This unit focuses on disrupting disordered eating behaviors and includes psychoeducation about meal planning, healthy exercise habits, dieting, binge eating, and purging. Users begin to monitor weight, monitor triggers, and conduct behavioral chain analyses. Self-care and social support are also included.
**Body Image**	11-15	This unit begins with psychoeducation and practice of body image tracking to help users become aware of thoughts and behaviors that reinforce poor body image. Users are led through exercises that help reveal the relative importance of body image in self-esteem so that they are empowered to re-prioritize. The core cognitive interventions in CBT are reviewed and users are provided rationale for targeting thought processes to reduce disordered eating behaviors and body image concerns. The user completes a series of techniques to gain greater awareness of automatic thoughts that may contribute to eating disorder thoughts/behaviors and low mood and practices reframing problematic thoughts. Users learn about cultural and media influences on body ideals and articulate a healthy ideal that can be used to replace the harmful cultural ideal. The concept of body avoidance is addressed and users complete a mirror exercise to break avoidance patterns.
**Behavior Change**	16-20	The behavior change unit helps the user identify forbidden foods that they restrict or avoid and plan the re-introduction of these foods into a healthy, balanced diet. Relaxation techniques and planning enjoyable activities are included to help users learn positive coping skills.
**Emotion Regulation**	21-25	This unit helps the user identify their emotions and how to cope with them using self-care and mindfulness techniques. User gains access to the emotion tracker.
**Shape Checking and Avoidance**	26-28	This unit is focused on helping the user understand the relationship between shape checking and avoidance behavior and reinforcement of negative body image thoughts. Users track shape checking and avoidance and are taught to disrupt these behavioral patterns. Users reflect on comparisons and learn to apply self-compassion concepts.
**Automatic Thoughts**	29-30	This unit helps the users identify automatic thoughts and provides an overview of the most common logical errors. Users are encouraged to track their thoughts using the automatic thought tracking tool.
**Cognitive Reframing**	31-35	This unit focuses on the cognitive reframing technique. Users learn how to reframe their negative thoughts including their self-critical thoughts.
**Habit Formation**	36-40	This unit teaches relapse prevention with a focus on maintaining the healthy habits users have created through the program and identifying triggers. Users are introduced to the to a tool to help them identify possible challenges ahead brainstorm solutions.

© 2020 Fitzsimmons-Craft EE et al. *JAMA Network Open.*

eTable 2. Frequencies of Possible Diagnoses at Post-intervention and Follow-up Time Points[a]

	**Intervention Condition**	**Control Condition**
**Post-intervention, *n* (%)**		
No diagnosis	69 (28.7%)	68 (27.1%)
Anorexia nervosa (AN)	2 (0.8%)	0 (0.0%)
Bulimia nervosa (BN)	19 (7.9%)	28 (11.2%)
Binge-eating disorder (BED)	15 (6.3%)	22 (8.8%)
Subthreshold BN	62 (25.8%)	67 (26.7%)
Subthreshold BED	47 (19.6%)	35 (13.9%)
Purging disorder	6 (2.5%)	7 (2.8%)
Unspecified feeding or eating disorder	19 (7.9%)	24 (9.6%)
**1-year follow-up, *n* (%)**		
No diagnosis	96 (39.0%)	79 (32.1%)
AN	0 (0.0%)	1 (0.4%)
BN	17 (6.9%)	23 (9.3%)
BED	12 (4.9%)	13 (5.3%)
Subthreshold BN	44 (17.9%)	56 (22.8%)
Subthreshold BED	48 (19.5%)	38 (15.4%)
Purging disorder	6 (2.4%)	9 (3.7%)
Unspecified feeding or eating disorder	20 (8.1%)	27 (11.0%)
**2-year follow-up, *n* (%)**		
No diagnosis	127 (49.8%)	115 (47.5%)
AN	1 (0.4%)	1 (0.4%)
BN	10 (3.9%)	16 (6.6%)
BED	8 (3.1%)	13 (5.4%)
Subthreshold BN	37 (14.5%)	47 (19.4%)
Subthreshold BED	48 (18.8%)	26 (10.7%)
Purging disorder	9 (3.5%)	9 (3.7%)
Unspecified feeding or eating disorder	14 (5.5%)	14 (5.8%)

Note. [a]Diagnosis based on the Stanford-Washington University Eating Disorder Screen (SWED).

© 2020 Fitzsimmons-Craft EE et al. *JAMA Network Open.*
