# Supplementary material for: Effectiveness of a Digital Cognitive Behavior Therapy–Guided Self-Help Intervention for Eating Disorders in College Women: A Cluster Randomized Clinical Trial
Source: JAMA Netw Open. 2020 Aug 31;3(8):e2015633. doi: 10.1001/jamanetworkopen.2020.15633 (PMC7489868; doi:10.1001/jamanetworkopen.2020.15633)
Supplement: Supplement 3. — Data Sharing Statement [file jamanetwopen-e2015633-s003.pdf]

Data Sharing Statement

Fitzsimmons-Craft. Effectiveness of a Digital Cognitive Behavior Therapy-Guided Self-Help Intervention for Eating Disorders in College Women. *JAMA Netw Open*. Published August 31, 2020. 10.1001/jamanetworkopen.2020.15633

Data
Data available: Yes
Data types: Deidentified participant data
How to access data: Data sharing statement: Data Data available: Yes Data types: Deidentified participant data that underlie the results reported in this article (texts, tables, figures, appendices). How to access data: Request from contact author (wilfleyd@wustl.edu) When available: Beginning 12 months following article publication; no end date. Supporting Documents Study protocol, statistical analysis plan. Additional Information Who can access the data: Researchers whose proposed use of the data has been approved by the PIs of the project. Types of analyses: If requests are within the scope of the research team to support so that appropriate analyses can be conducted, a data sharing agreement will be established between the institutions (and investigators) requesting and holding the research data. Mechanisms of data availability: Proposals and request for data should be directed to the contact author. A data sharing agreement will also be established.
When available: beginning date: 04-01-2021

Supporting Documents
Document types: None

Additional Information
Who can access the data: Researchers whose proposed use of the data has been approved by the PIs of the project.
Types of analyses: If requests are within the scope of the research team to support so that appropriate analyses can be conducted, a data sharing agreement will be established between the institutions (and investigators) requesting and holding the research data.
Mechanisms of data availability: Proposals and request for data should be directed to the contact author. A data sharing agreement will also be established.
Any additional restrictions: n/a
